# Evolution of MHC-based technologies used for detection of antigen-responsive T cells

**DOI:** 10.1007/s00262-017-1971-5

**Published:** 2017-03-17

**Authors:** Amalie Kai Bentzen, Sine Reker Hadrup

**Affiliations:** Section for Immunology and Vaccinology, National Veterinary Institute, Technical University of Denmark, Copenhagen, Denmark

**Keywords:** MHC multimer, T cell receptor, MHC class I, Antigen specificity, DNA barcode-labeled MHC multimers

## Abstract

T cell-mediated recognition of peptide-major histocompatibility complex (pMHC) class I and II molecules is crucial for the control of intracellular pathogens and cancer, as well as for stimulation and maintenance of efficient cytotoxic responses. Such interactions may also play a role in the development of autoimmune diseases. Novel insights into this mechanism are crucial to understanding disease development and establishing new treatment strategies. MHC multimers have been used for detection of antigen-responsive T cells since the first report by Altman et al. showed that tetramerization of pMHC class I molecules provided sufficient stability to T cell receptor (TCR)-pMHC interactions, allowing detection of MHC multimer-binding T cells using flow cytometry. Since this breakthrough the scientific community has aimed for expanding the capacity of MHC multimer-based detection technologies to facilitate large-scale epitope discovery and immune monitoring in limited biological material. Screening of T cell specificity using large libraries of pMHC molecules is suitable for analyses of T cell recognition potentially at genome-wide levels rather than analyses restricted to a selection of model antigens. Such strategies provide novel insights into the immune specificities involved in disease development and response to immunotherapy, and extend fundamental knowledge related to T cell recognition patterns and cross-recognition by TCRs. MHC multimer-based technologies have now evolved from detection of 1–2 different T cell specificities per cell sample, to include more than 1000 evaluable pMHC molecules using novel technologies. Here, we provide an overview of MHC multimer-based detection technologies developed over two decades, focusing primarily on MHC class I interactions.

## Introduction

**Fig. 1:**
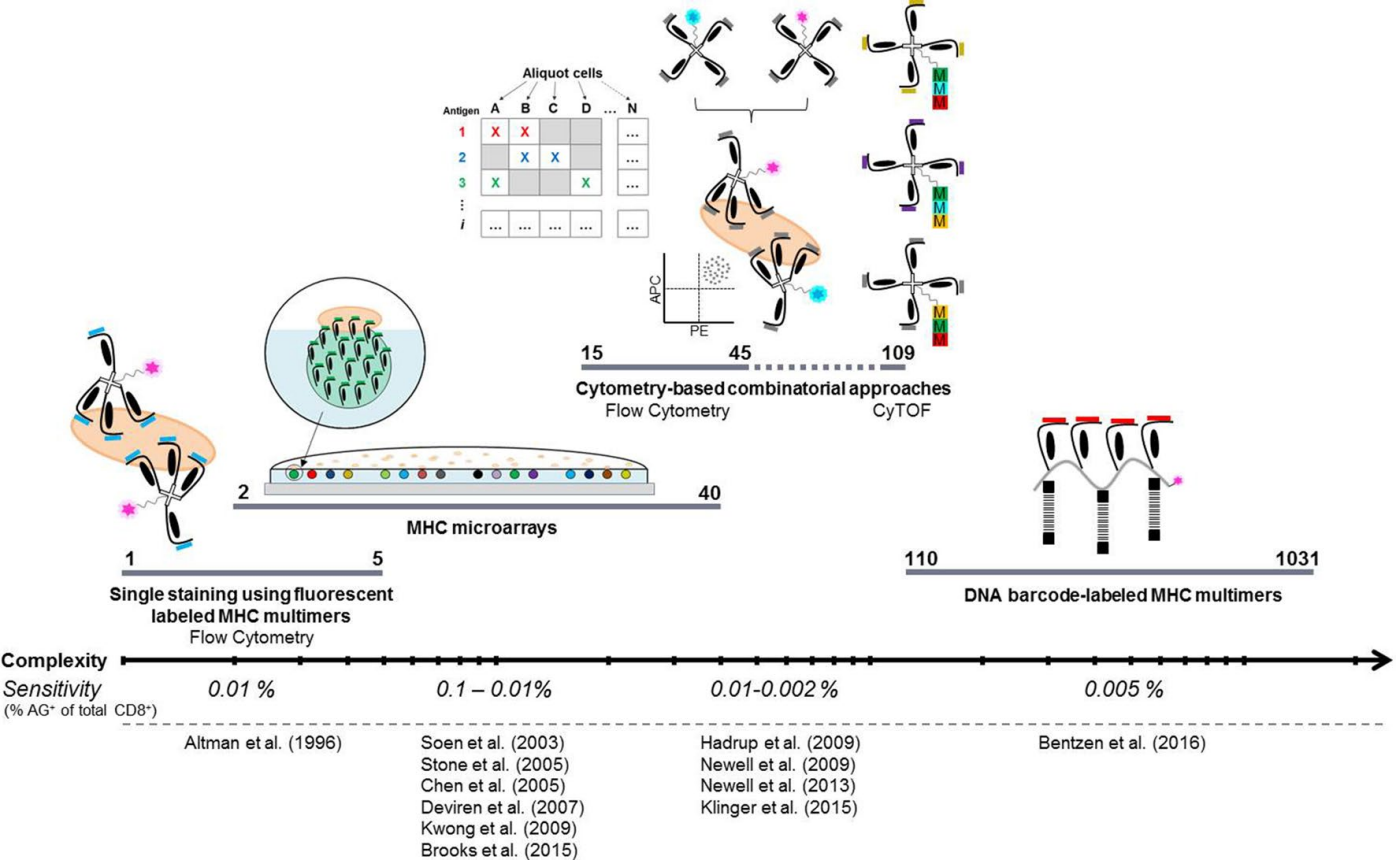
Evolution of MHC-based detection of antigen-responsive T cells. The methodologies are shown on a Log scale with increasing complexity in terms of the reported number of distinct antigen-responsive T cell populations that may by identified per sample. Most of the strategies offer the potential to screen for T cell reactivity at higher complexities. The listed sensitivities are based on screening for antigen-specific recognition among 2 × 10^6^ viable cells, though the mass cytometry and matrix-based approaches require a higher number of input cells. The DNA barcode-based methodology has been reported to have an increased sensitivity with an increased number of input cells. *AG* ^*+*^ antigen positive, *CyTOF* cytometry by time-of-flight

Techniques for the detection of antigen-responsive T cells exploit the interaction between a given TCR and its pMHC recognition motif. T cell detection using multimerized pMHC molecules has evolved into the preferred method for detecting antigen-specific T cells. Over the past 20 years, since the first multimerizations of pMHC molecules [1], several approaches have aimed at matching the heterogeneity of T cells and epitope presentation to enhance our ability to map immune responses. The evolution of these approaches, in terms of increasing complexity, is shown in Fig. 1.

MHC multimer technologies have primarily been developed and applied for analyses of CD8 T cell responses, because MHC class I molecules have proven easier to handle in terms of protein folding and expression. Additionally, the MHC class I binding groove is more restricted in terms of the length of peptide bound—hence, it is more straightforward to predict MHC class I binding peptides. Most of the technologies described in this review relate to detection of specific CD8 T cell responses, but they are in principle also applicable to MHC class II multimers and detection of CD4 T cell responses. Specific challenges associated with the production and use of MHC class II multimers are addressed in the final section.

MHC molecules are largely unstable when they are not part of a complex with peptide. For this reason, pMHC-based technologies were initially restricted by the tedious production of pMHC molecules, where each peptide required an individual folding and purification procedure [2, 3]. Thus, the development of high-throughput strategies for T cell identification was constrained by the limiting step involving the generation of large libraries of pMHCs. A number of potential solutions to this challenge have been developed in the last decade. First, Schumacher et al. described the use of conditional MHC ligands that are cleaved upon exposure to 366 nm UV-light and can be exchanged with any MHC ligand of interest [4]. Using this strategy, individual MHC class I molecules are correctly refolded with carefully designed UV-cleavable peptides (p*), allowing sufficient stability of the complex. Individual p*MHC molecules are purified, and stored to serve as a source of stock molecules that can be exchanged with any ligand of interest upon exposure to UV-light. The UV-cleavable conditional ligand-strategy has enabled the production of large numbers of different pMHC molecules in a high throughput manner [5, 6]. Today, such UV-ligands have been designed for a number of different MHC class I alleles, of both human and murine origin [7, 8]. An alternative strategy is the preferential folding of correctly oxidized MHC class I heavy chains. This allows efficient folding-reactions in small volumes, reduces the need for further optimization and can be used to create large libraries of diverse pMHC complexes [9]. More recently, it was discovered that certain di-peptides can assist folding and peptide exchange of MHC class I molecules [10, 11]. Di-peptides bind specifically to the F pocket of MHC class I molecules to facilitate peptide exchange and have so far been described and validated for peptide exchange in HLA-A*02:01, HLA-B*27:05, and H-2K^b^ molecules. The di-peptide exchange technology has not yet been applied in larger T cell epitopes mapping strategies. Together, these technologies have enabled efficient production of large libraries of pMHC molecules, and consequently high-throughput detection of CD8 T cell recognition using pMHC-based reagents.

## Strategies for high-throughput detection of antigen-responsive T cells

All the MHC-based strategies described throughout this report are summarized in Table 1.

**Table 1:** Summary of multiplex MHC-based strategies for detection of antigen-responsive T cells

Strategy for “high-throughput” detection of specific CD8 T cell responses	References	Reported sensitivity (frequency of specific T cells)	Reported order of complexity	Combined with functional and/or phenotypic readout	Offer recovery of AG specific T cells
DNA barcode labeling of pMHC multimers Detection by sequencing	Bentzen et al. [28]	0.005% of CD8^+^ T cells	1031 different specificities per sample (0.20 × 10^6^–10 × 10^6^ PBMCs)	Yes	Yes
Three-dimensional combinatorial encoding of metal labeled pMHC multimers Detection by cytometry by time-of-flight	Newell et al. [25]	0.001% of CD8^+^ T cells (after MHC tetramer-based enrichment)	109 different specificities per sample (16 × 10^6^–302 × 10^6^ PBMCs)	Yes	No
Two-dimensional combinatorial encoding of fluorescent labeled pMHC multimers Detection by flow cytometry	Andersen et al. [31], Hadrup et al. [20]	0.002% of CD8^+^ T cells	36 different specificities per sample (2 × 10^6^ PBMCs)	Yes	Yes
Combinatorial pooling of fluorescent labeled pMHC multimers and splitting of sample material into each pool Detection by flow cytometry	Klinger et al. [27]	Not reported	16 different specificities per analysis^a^	No^a^	Yes
Multi-dimensional combinatorial encoding of fluorescent labeled pMHC multimers Detection by flow cytometry	Newell et al. [21]	0.01% of CD8^+^ T cells	15 different specificities per sample (2 × 10^6^ PBMCs)	No	Yes
MHC microarray: pMHC tetramers spotted onto polyacrylamide slides combined with an aggressive washing scheme	Brooks et al. [19]	0.02% of CD8^+^ T cells (after CD8^+^ T cell enrichment)	40 different specificities per assay (0.8–1.2 × 10^6^ T cells)	No	No
MHC microarray: DNA-probed pMHC tetramers spotted onto complementary DNA probe printed glass slide	Kwong et al. [18]	0.1% of CD8^+^ T cells	3 different specificities per assay (10^6^ T cells)	No	Yes
MHC microarray: Dimeric pMHCs spotted onto antibody-coated polyacrylamide microscopy slide	Deviren et al. [17]	0.01% of CD8^+^ T cells	2 different specificities per assay (10^6^ cells)	No	No
MHC microarray: Dimeric pMHCs co-spotted with cytokine capture antibodies onto polyacrylamide microscopy slide	Chen et al. [16]	0.01% of CD8^+^ T cells	7 different specificities per assay (CD8^+^ T cells isolated from 5 × 10^7^ PBMCs)	Yes	No
MHC microarray: pMHCs co-spotted onto polystyrene microscopy slide with cytokine capture antibodies and co-stimulatory molecules	Stone et al. [15]	0.1% of CD8^+^ T cells	30 different specificities per assay (10^6^ cells)	Yes	No
MHC microarray: pMHCs tetramers spotted onto polyacrylamide microscopy slide	Soen et al. [14]	0.1% of CD8^+^ T cells	7 different specificities per assay (10^6^ cells)	Yes (Ca^2+^ levels)	No

^a^A non-MHC variation of this approach based on activation of T cells has been applied to screen for 30 specificities in parallel

### Detecting antigen-responsive T cells using MHC microarrays

Early attempts to improve the complexity of MHC multimer-based detection of antigen-responsive T cells were based on the success of gene-expression arrays, including the availability of efficient systems capable of quantifying fluorescent probes when spatially separated as spots on a microarray [12]. Protein-based microarrays have been developed [13] and the idea of a pMHC microarray allowing cell-capture based on specificity is appealing. Although several such systems have been developed [14–19], their application seems to be limited on account of a poor detection limit and low reproducibility compared to existing cytometry-based analyses. There is considerable overlap between MHC microarray approaches, where most work has focused on optimizing the supporting surface and modifying the conditions applied during binding and/or washing.

In early studies by Soen et al. [14] and Chen et al. [16], microscope slides coated with polyacrylamide were selected as the optimal surface to lower cellular interaction while aiding protein binding. In these studies, the researchers were able to screen for seven different antigen-specificities in parallel and detect populations of T cells at a minimum frequency of 0.1% of total CD8 T cells [14] or, sensitivity may be enhanced up to tenfold when isolating CD8 T cells prior to loading them onto the microarray [16]. Using similar approaches, Stone et al. [15] and Brooks et al. [19] showed a considerable increase in complexity, screening for 30–40 different antigen-responsive T cells in parallel, while also achieving a detection limit of approximately 0.1% of CD8 T cells. Using microarray approaches pMHC molecules can be co-spotted either with cytokine capture antibodies [16] or co-stimulatory molecules [15]. This may provide additional functional information to the T cell identification process. Moreover, the visualization of activation markers once the T cells are captured at pMHC specific spots may improve the sensitivity of the assay [15, 16]. The study by Chen relied solely on T cell activation from multimeric pMHC:TCR interaction; however, multimeric pMHC:TCR interaction cannot be expected to activate all relevant T cells. Thus, counting on a lower detection limit due to the capture and visualization of activation markers would also increase the risk of missing those T cell populations that were not activated in such a system or misinterpreting their frequency.

A general limitation with such array-based strategies is the propensity of a given T cell to pursue all potential pMHC interactions displayed on a given array. As a consequence, the frequency of antigen-responsive T cells in the cell preparations should be >0.1% to allow a robust readout. Deviren et al. [17] particularly addressed the issue of assay reproducibility and homogeneity, and by introducing a mild flow shear they obtained a more uniform distribution of captured T cells and a higher reproducibility. Consistent with the issue of ensuring cell interaction at all possible pMHC positions, they noted a greater variability associated with decreased surface area of each spotted pMHC. The nucleic acid cell sorting (NACS) approach developed by Kwong et al. [18] employed unique sequences of single-stranded DNA conjugated to distinct pMHC tetramers, which in turn were site-specifically assembled via hybridization onto glass slides printed with complementary DNA probes. This strategy offers an advantage over other MHC arrays in that T cells can be recovered from the glass slide by selective detachment using restriction enzymes.

### Detecting antigen-responsive T cells using combinatorially labeled MHC multimers

An alternative approach to pMHC microarrays is to utilize the increasing number of fluorescent labels available for flow cytometry. Two such parallel approaches were described in 2009, using combinatorial encoding of fluorescently labeled MHC multimers. Both of these approaches take advantage of the assumption that any given T cell in the cell samples will only bind one of the pMHC molecules in a given library. Thus, each distinct pMHC multimer can be assigned with a unique dual color code [20] or multivalent code [21], which is then used to identify the T cells binding to this particular pMHC molecule. When employing the unique dual color code, any T cell not matching the pre-determined color combinations is recorded as a background event. This provides a low limit of detection (0.002%) and a clear distinction between background events and specific pMHC binding with T cells. However, both approaches require that peptide sequences with minimal variation are analyzed in separate samples, to avoid cross-recognition between peptides within a given sample.

With the emergence of mass cytometry that uses heavy metal ions as tags, there has been a dramatic leap in the number of labels that can be applied in parallel [22–24]. Combining mass cytometry with combinatorial pMHC tetramer staining using a total of 10 metal labels applied in unique combinations of three labels per MHC multimer, Newell et al. were able to screen for 109 different antigen-specificities in one sample, while still leaving a substantial number of labels for parallel phenotype and functional analysis of T cells [25]. Despite the obvious progress made using this assay, there are a number of drawbacks associated with mass cytometry which may have impeded the widespread use of the method. This includes low cell recovery in the instrument (~30%), slow collection speed (~300 events/s), relatively low resolution, and high instrument and reagent cost [26]. Most importantly, the instrument offers no way of recovering distinct populations, as the cell structure is fully disrupted during the process. Thus, potential combination with genomic or transcriptomic sequencing-based approaches is not feasible.

More recently, a matrix-based approach has been developed for T cell identification. In this method, a set of MHC multimers are split into matrix-defined pools, in such a way that no specific MHC multimer are present in all pools. The multimer pools are used to stain an equal number of cell aliquots derived from a given sample. The approach uniquely combines antigen recognition with sequencing of the TCRβ chain in that the MHC multimer positive and negative populations of each reagent pool are sorted and clonotypes enriched within the positive population are identified [27]. The advantage of this approach is that, theoretically, the number of antigen-responsive T cells that can be assessed in parallel increases exponentially with the number of pools created. In practice, 16 antigen-specificities have been analyzed in parallel (8 pools with 4 different MHC multimers per pool). The same study also uses an alternative, non-MHC-based approach that applies stimulation with different pools of peptides and sorting of the activated subset of T cells to screen for recognition of 30 peptide-antigens in parallel.

This forms an attractive tool for detecting many antigen-responsive T cells simultaneously, especially since it also enables the identification of the corresponding TCRβ clonotypes. However, the necessity to split samples into a number of pools represents an inherent drawback in terms of detecting rare T cell populations especially in patient material that is often of limited access.

Thus, until recently no technology has been available that simultaneously permits high-throughput analyses of T cells while tolerating the large diversity of T cell antigen specificity, thereby allowing detection of epitopes at potentially genome wide levels even in limited biological samples, and recovery the positively interacting T cells for gene expression analysis.

## Next generation detection of antigen-responsive T cells using DNA barcode-labeled MHC multimers

**Fig. 2:**
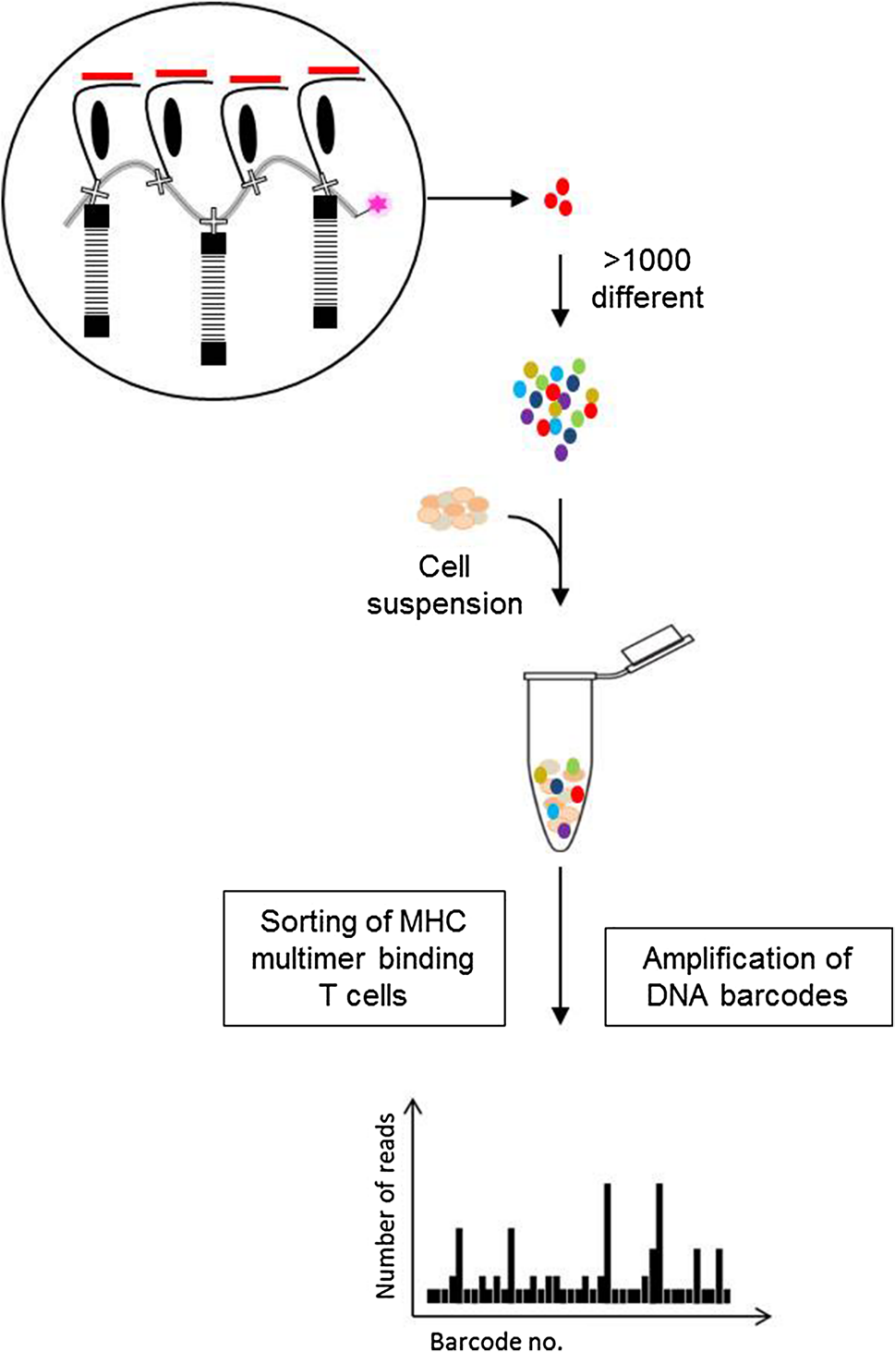
Detection of antigen-responsive T cells using the DNA barcode-labeled MHC multimer methodology. A PE-labeled dextran backbone carrying a number of SA binding sites (illustrated as an *X*) is applied to co-attach biotinylated molecules; DNA barcodes and pMHCs. Thus >1000 pMHC multimers can be generated, each carrying a different DNA barcode. All MHC multimer binding T cells are sorted based on the common fluorescent label and the associated DNA barcodes are amplified and sequenced. The relative numbers of DNA barcode reads are used to determine the composition of antigen-responsive T cells in the sample

The most recent addition to the immunologist toolbox for detecting T cells based on antigen recognition involves implementation of a new type of label. Instead of using fluorescence or metal labels, as described in previous sections, we [28] have applied unique DNA barcodes that when attached to MHC multimers, form specific tags for the given pMHC epitopes. DNA barcodes can be designed with a complexity of up to 10^10^ different sequences, each forming a unique tag [29]. As a result, this strategy is not limited by the number of available labels, which has previously been the most critical barrier to high-throughput detection of antigen-responsive T cells. We have shown that this approach can be applied to screen for more than 1000 specificities in a single sample. These MHC multimers are generated on a backbone of polysaccharide dextran that carries a number of free streptavidin (SA) binding sites. These SA binding-sites are used to co-attach biotinylated molecules, recombinant pMHCs, and DNA barcodes (Fig. 2). In this manner, every pMHC epitope is associated with a unique DNA barcode (e.g., barcode#1 codes for pMHC#1, barcode#2 codes for pMHC#2 and so on) that can be easily recovered by high-throughput sequencing. Moreover, all MHC multimers are conjugated to the same fluorochrome (PE), whose signal-intensity serves as a means to collectively sort any T cells that associate with the MHC multimers. Thus, first the T cells are incubated with large collections (>1000) of DNA barcode-labeled MHC multimers and all multimer positive cells are sorted, and then the associated DNA barcodes are amplified and the unique sequences revealed after sequencing (Fig. 2). Ultimately, the number of reads for a specific DNA barcode matching the corresponding pMHC correlates with the presence of antigen-responsive T cells within the sorted population.

In contrast to both single MHC multimer staining and combinatorial encoding of MHC multimers (using either fluorescence or metal labels) where the signal intensities are applied directly to define the antigen recognition of a given T cell population, the antigen-responsiveness determined using DNA barcode-labeled MHC multimers are defined in a retrospective analysis of sequence reads, providing an estimate for the number of antigen-responsive T cells rather than the exact number. Unique molecular identifiers [30] are incorporated in the DNA barcode design to provide a means of accounting for amplification bias, and thus the number of sequence reads reflects the number of specific pMHC:TCR interactions in the population as a whole. In other words, when calculating the frequency of antigen-responsive T cells based on the number of barcode reads and the frequency of total multimer-positive cells, the determined frequencies are based on an average number of pMHC:TCR interactions for all T cells in the potentially heterogeneous population. This strategy may introduce a bias in frequency determination, depending on pMHC:TCR affinity and TCR expression level on the given cell population. Practically, this phenomenon seems to provide limited variability to the DNA barcode-based determination of T cell frequencies, resulting in a very tight correlation with T cell frequencies determined by combinatorially encoded fluorescently labeled MHC multimers [*r* ^2^ = 0.967, across 10 healthy donors (32 responses) and *r* ^2^ = 0.901 across 11 melanoma patients (15 responses)] [28].

The DNA barcode-based approach offers the advantage that it is less dependent on stringent fluorescent-based separation of multimer-positive from multimer-negative events, because even events that may not be fluorescently separated due to low level pMHC multimer interactions will still bind enough DNA barcode-labeled MHC multimers to allow recovery of their specific DNA barcode element, thus enabling the identification of the TCR specificity. The technology therefore represents an attractive tool for detecting low avidity TCR:pMHC interactions such as shared tumor-associated antigens. When applying cytometry-based approaches, the signal intensity associated with a given pMHC is the only measure distinguishing those T cells that interact specifically with MHC multimers and those that do not. Therefore, it is essential that a given T cell binds numerous MHC multimers, to obtain sufficient fluorescence intensity and thereby enable unambiguous identification of their specificity. A T cell that has low affinity TCR:pMHC interactions or low surface TCR expression may not bind sufficient MHC multimers to obtain fluorescent separation, but will still provide a signal from the attached DNA barcode. We have demonstrated that the antigen-responsive composition of T cells in a sample can be determined independent of any fluorescent label, by sorting the whole CD8 T cell population as opposed to only the multimer-positive T cells [28].

In line with the reasoning that antigen recognition can be determined from the number of DNA barcode reads irrespective of the fluorescent-based separation of multimer-positive cells, other criteria can be applied to determine which cell populations to sort. Throughout the workflow of the DNA barcode-based strategy only a few different fluorescent labels are applied to determine the viable CD8 positive population, leaving a considerable number of fluorescent labels to stain for other cell properties. Thus, one can distinguish between CD8 T cells with certain phenotypic characteristics or of a certain activation status, and retrospectively determine the composition of antigen-responsive T cells among the different populations through sequencing of the associated DNA barcodes. This strategy was illustrated with the activation of antigen-responsive T cells present in healthy donor blood or tumor-infiltrating lymphocytes (TILs), through stimulation with a pool of common virus-derived peptides or autologous tumor cell line, respectively. Subsequently, samples are stained with collections of DNA barcode-labeled MHC multimers and intracellular activation markers. The fluorescent signal from the MHC multimers was disregarded and cells were sorted exclusively based on the cytokine secretion profile [28]. Thus, we have shown that DNA barcodes associated with pMHC multimers recognized by T cells that have been exposed to their target antigen, can completely or predominantly be recovered from the activated subset of CD8 T cells. On the other hand, the DNA barcodes associated with epitopes not available for T cells during stimulation are only found in the non-activated subset of CD8 T cells. TCR downregulation following T cell activation has previously conflicted with effective combinations of MHC multimer staining and T cell activation for low-avidity T cells; however, the DNA barcode-labeled strategy seems to be less sensitive to such phenomena, because even low levels of pMHC multimer interactions (insufficient for fluorescent separation) will retain a DNA barcode signal, as described above [28].

Inarguably, the greatest advantage of using DNA barcode-labeled MHC multimers is the possibility to screen in an unbiased manner for T cell reactivity across large libraries of pMHC epitopes in a single sample. Applying the current DNA barcode design, we can generate unique labels in the order of 10^10^; thus the technology has the potential for analyzing T cell reactivity at close to genome-wide levels. Using earlier high-throughput methodologies it has not been possible to analyze for T cell reactivity of structurally related pMHC epitopes in the same sample, e.g., peptides differing only at a single amino-acid position or 9-, 10- or 11-mer versions of the same peptide with a given HLA restriction. However, this issue could be circumvented without much difficulty since these strategies, being only semi-high-throughput, have often required the splitting of samples into several tubes to analyze all potential epitopes of interest. Users simply have to ensure that different versions of a pMHC epitope were represented in different MHC reagent mixtures. However, with the possibility of screening, e.g., a full cancer-mutagenome in one sample, it is essential that structurally related pMHC epitopes can be identified in parallel using only one mixture of MHC reagents. The DNA barcode-based approach provides information on recognition of a given pMHC molecule, but such recognition may likely be shared among several T cell populations. Thus, cross reactivity to multiple epitopes in a given library will not disrupt analyses. Analyzing the cross-reactive features and relative recognition profile among different pMHC complexes can be accomplished when T cells are assessed at a single cell level.

The DNA barcode labeling of MHC multimers provides a new tool to gain insight to T cell recognition in complex disease situations. Nonetheless, the main disadvantage of the methodology is that it merely provides an estimate for T cell frequencies, and does not offer any possibility for direct visual assessment of the findings in terms of T cell recognition. Thus, users rely on good quality assurance of the sequencing procedure and validated programs to translate read number into the presence of antigen-responsive T cells. Furthermore, pMHC binding T cells are lysed in the process of DNA barcode amplification, and are consequently not available for subsequent cell culturing.

## Novel insights into T cell recognition of cancer

Due to the limited number of cells available directly from excised tumor fragments, it has for most prior purposes been necessary to expand TILs in vitro to enable analyses of antigen recognition of the relevant pMHC multimer molecules. Previous studies have shown that expansion of T cells may introduce a bias in antigen recognition due to variable growth potential of TILs, and specifically, preferential growth of T cell populations that recognize virus epitopes [31]. Application of large-scale screening strategies reduces the need for such in vitro expansion of T cells, and allows for T cell recognition analyses on broad spectra of pMHC epitopes even in minute amounts of patient material. We have shown the possibility for detection of antigen-responsive T cells towards a large shared melanoma library (composed of 175 DNA-barcoded MHC multimers) in tumor digest from melanoma patients without the need for T cell expansion [25]. Moreover, because of the amount of data that can be recovered from a relatively small sized sample, the DNA barcode-based methodology represents an attractive tool to analyze patient samples taken at multiple time-points, to follow the dynamics of an immune response and the response to certain treatments.

Since it is no longer necessary to split samples into multiple aliquots to screen for antigen recognition, it is possible to look for T cell recognition in an unbiased fashion in samples with a considerably greater number of precursor cells than what had previously been applied. With increasing cell numbers, the stochastic variation of the compositions of antigen-specific T cell populations is reduced, and the chance for detecting low-frequent T cell populations increases, reflecting a higher absolute number of pMHC responsive T cells in the given sample.

The advantages of being able to include more T cells in the initial binding reaction were illustrated in a study where we screened for T cell recognition of predicted HLA-binding peptide sequences containing cancer-specific mutations in two patients with non-small cell lung carcinoma. A large number of personal neoepitope peptides had been predicted for each patient resulting in libraries of 288 and 417 different pMHCs, respectively [32]. In these patients, T cell recognition was assessed from analyzing in vitro expanded TILs from one or more regions of lung tissue. A total of one neoepitope-responsive T cell population was detected using combinatorial encoding of fluorescent labels, which allowed the inclusion of only 1 × 10^6^ TILs/MHC reaction mixture (9–13 different reaction mixtures/patient specific neoepitope library). Despite the limited availability of sample, we were able to detect a total of nine different neoepitope-responsive T cells when applying the DNA barcode-based MHC multimer technology. This may partly be explained by the greater number of cells included in the initial binding reactions (4–8 × 10^6^ TILs in one MHC reaction mixture) and due to the possibility for analyzing peripheral blood. In fact, several of the detected responses were predominantly or exclusively found in the peripheral blood sample [28].

## MHC class II multimers for detection of antigen-responsive CD4 T cells

An additional challenge in the field of immune analyses relates to the characterization of antigen-responsive CD4 T cells. Antigen-responsive CD4 T cells are believed to play a significant role in tumor-cell recognition [33, 34] and development of autoimmune diseases [35, 36]. Detection of antigen-specific CD4 T cells using MHC II multimers is feasible [37], but high-throughput detection strategies are still challenging. MHC class II molecules require, for most alleles, expression in mammalian cells [38], although more recent strategies has proven that functional HLA-DR molecules can be expressed and refolded from *E. coli* [39]. Peptide exchange can be facilitated using constructs that includes a thrombin cleavage site next to a class II-associated invariant chain peptide (CLIP). MHC II are produced with CLIP embedded in the peptide binding groove, but following thrombin-based cleavage of the linker, CLIP can be displaced by simple competition with ligands of interest. This strategy has been exploited to generate small libraries of MHC II molecules, DRB1*0101, DRB1*0401, DRB5*0101 and DRB1*1501 [40]. Importantly, it is much more difficult to predict which peptides will form stable complexes with MHC class II compared to class I since the open structure of the MHC II molecule enables peptide fragments to protrude from the binding groove and thus allows much more variable side chains of cargo peptides [41]. Moreover, it is presumed that CD4 TCRs bind with lower affinity to their cognate pMHC epitope than CD8 TCRs [42]. This may be a particularly important limitation in the ability to detect self-antigen and tumor-specific CD4^+^ T cell populations.

Along with the improvements in prediction tools for MHC-II ligands [43] and the development of efficient peptide replacement systems for generating large libraries of MHC class II monomers [40], the technologies for large-scale detection of antigen-responsive CD8 T cells described herein should also be feasible for detection of CD4 T cells. Specifically, the DNA barcode-based methodology may provide an advantage, compared to the other methods, for detection of CD4 T cells using MHC II multimers, due to its particular advantages in terms of detecting T cells with low-affinity TCR:pMHC interactions, that are not well separated based on fluorescence labeling alone. However, the use of DNA barcode labeled MHC II multimers has not yet been investigated.

## Future implications

At present proof-of-principle have been provided that up to 1031 DNA barcode-labeled MHC multimers can be applied in one reaction mixture to detect several populations of antigen-responsive T cells in parallel. This complexity, though already representing a tenfold leap compared to earlier methodologies, can potentially be increased to >10,000–100,000 differently labeled MHC multimers. Since the potential complexity of the DNA barcode label is in the order of 10^10^, the limitations inherent to combinatorial encoding using fluorescent or metal tags have been removed. However, new bottlenecks may arise with increasing library sizes. Moving the complexity of MHC multimer libraries within the range of 10,000–100,000 specificities will require automation of all aspects of the pMHC multimer production as well as more efficient and cost-effective production of synthetic peptides. Furthermore, it will most likely require the development of microfluidics systems that can ensure efficient pMHC interaction with all T cells in a given sample.

**Fig. 3:**
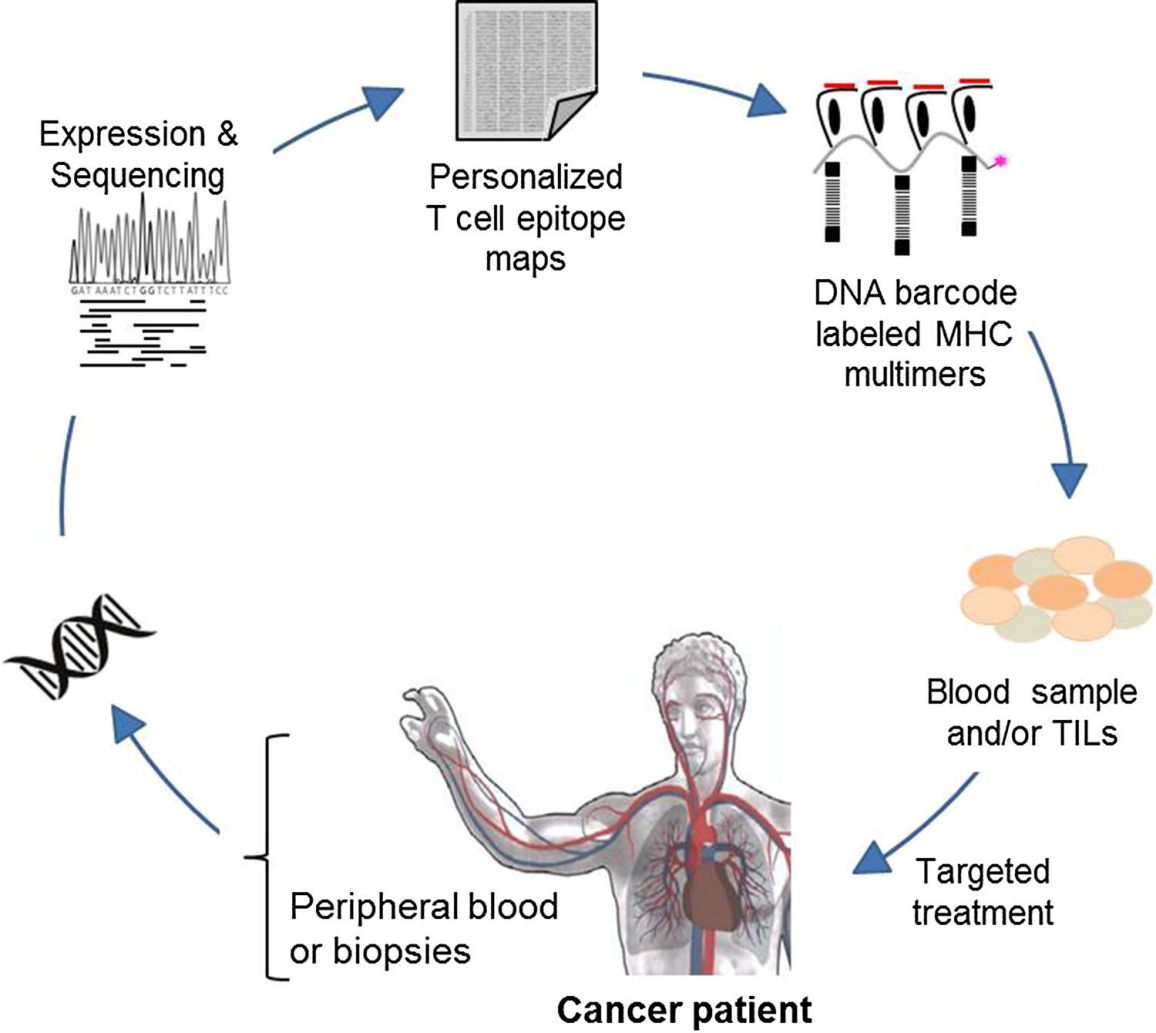
Detection of neoepitope-responsive T cells in cancer patients. T cell responses in cancer patients rely to a large extent on epitope recognition defined by both expression and mutational patterns given in the individual patient’s tumor. Targeting of mutation-derived neoepitopes in cancer therapy requires the identification of personalized epitope maps and prediction or preferably testing for immunogenicity. High-throughput strategies for T cell analyses will play an important role in this process

In the era of personalized medicine and with accumulating types of immunotherapeutic strategies being employed, it is becoming increasingly important to understand the T cell-mediated recognition of cancer cells, and identify biomarkers predicting the response to therapy—potentially based on experimental analyses of T cell recognition (Fig. 3).

Recent data suggest that a substantial fraction of the T cell reactivity induced during checkpoint inhibition is directed towards neoepitopes that are generated through genetic alterations [44–46]. However, current tools for neoepitope prediction are imprecise and identification of tumor-rejection epitopes at genome-wide levels requires large-scale analyses of T cell reactivity. The recently developed technology based on DNA barcode-labeled MHC multimers has the potential to improve such T cell analyses, and importantly, enables detection of T cells in both peripheral blood and tissue biopsies [28]. Furthermore, increasing knowledge of the T cell recognition profile in cancer patients can be used to improve prediction tools for cancer-specific neoepitopes, and help in identifying the impact of genetic heterogeneity in T cell recognition [32].

## Abbreviations

AG^+^: Antigen positive
CLIP: Class II-associated invariant chain peptide
CyTOF: Cytometry by time-of-flight
NACS: Nucleic acid cell sorting
p*: UV-cleavable peptides
pMHC: Peptide-MHC
SA: Streptavidin

## References

[1] Altman JD, Moss PA, Goulder PJ (1996). Phenotypic analysis of antigen-specific T lymphocytes. Science.

[2] Bakker AH, Schumacher TNM (2005). MHC multimer technology: current status and future prospects. Curr Opin Immunol.

[3] Garboczi DN, Hung DT, Wiley DC (1992). HLA-A2-peptide complexes: refolding and crystallization of molecules expressed in Escherichia coli and complexed with single antigenic peptides. Proc Natl Acad Sci USA.

[4] Toebes M, Coccoris M, Bins A (2006). Design and use of conditional MHC class I ligands. Nat Med.

[5] Bakker AH, Hoppes R, Linnemann C (2008). Conditional MHC class I ligands and peptide exchange technology for the human MHC gene products HLA-A1, -A3, -A11, and -B7. Proc Natl Acad Sci USA.

[6] Rodenko B, Toebes M, Hadrup SR (2006). Generation of peptide-MHC class I complexes through UV-mediated ligand exchange. Nat Protoc.

[7] Frøsig TM, Yap J, Seremet T (2015). Design and validation of conditional ligands for HLA-B*08:01, HLA-B*15:01, HLA-B*35:01, and HLA-B*44:05. Cytom Part A.

[8] Chang CXL, Tan AT, Or MY (2013). Conditional ligands for Asian HLA variants facilitate the definition of CD8 + T-cell responses in acute and chronic viral diseases. Eur J Immunol.

[9] Leisner C, Loeth N, Lamberth K (2008). One-pot, mix-and-read peptide-MHC tetramers. PLoS One.

[10] Saini SK, Ostermeir K, Ramnarayan VR (2013). Dipeptides promote folding and peptide binding of MHC class I molecules. Proc Natl Acad Sci USA.

[11] Saini SK, Schuster H, Ramnarayan VR (2015). Dipeptides catalyze rapid peptide exchange on MHC class I molecules. Proc Natl Acad Sci USA.

[12] Schena M, Shalon D, Davis RW, Brown PO (1995). Quantitative monitoring of gene expression patterns with a complementary DNA microarray. Science.

[13] MacBeath G, Schreiber SL (2000). Printing proteins as microarrays for high-throughput function determination. Science.

[14] Soen Y, Chen DS, Kraft DL (2003). Detection and characterization of cellular immune responses using peptide-MHC microarrays. PLoS Biol.

[15] Stone JD, Demkowicz WE, Stern LJ (2005). HLA-restricted epitope identification and detection of functional T cell responses by using MHC-peptide and costimulatory microarrays. Proc Natl Acad Sci USA.

[16] Chen DS, Soen Y, Stuge TB (2005). Marked differences in human melanoma antigen-specific T cell responsiveness after vaccination using a functional microarray. PLoS Med.

[17] Deviren G, Gupta K, Paulaitis ME, Schneck JP (2007). Detection of antigen-specific T cells on p/MHC microarrays. J Mol Recognit.

[18] Kwong GA, Radu CG, Hwang K (2009). Modular nucleic acid assembled p/MHC microarrays for multiplexed sorting of antigen-specific T cells. J Am Chem Soc.

[19] Brooks SE, Bonney SA, Lee C (2015). Application of the pMHC array to characterise tumour antigen specific T cell populations in leukaemia patients at disease diagnosis. PLoS One.

[20] Hadrup SR, Bakker AH, Shu CJ (2009). Parallel detection of antigen-specific T-cell responses by multidimensional encoding of MHC multimers. Nat Methods.

[21] Newell EW, Klein LO, Yu W, Davis MM (2009). Simultaneous detection of many T-cell specificities using combinatorial tetramer staining. Nat Methods.

[22] Ornatsky OI, Baranov VI, Bandura DR (2006). Messenger RNA detection in leukemia cell lines by novel metal-tagged in situ hybridization using inductively coupled plasma mass spectrometry. Transl Oncogenomics.

[23] Bandura DR, Baranov VI, Ornatsky OI (2009). Mass cytometry: technique for real time single cell multitarget immunoassay based on inductively coupled plasma time-of-flight mass spectrometry. Anal Chem.

[24] Bendall SC, Simonds EF, Qiu P (2011). Single-cell mass cytometry of differential immune and drug responses across a human hematopoietic continuum. Science.

[25] Newell EW, Sigal N, Nair N (2013). Combinatorial tetramer staining and mass cytometry analysis facilitate T-cell epitope mapping and characterization. Nat Biotechnol.

[26] Spitzer MH, Nolan GP (2016). Mass cytometry: single cells, many features. Cell.

[27] Klinger M, Pepin F, Wilkins J (2015). Multiplex identification of antigen-specific T Cell receptors using a combination of immune assays and immune receptor sequencing. PLoS One.

[28] Bentzen AK, Marquard AM, Lyngaa R (2016). Large-scale detection of antigen-specific T cells using peptide-MHC-I multimers labeled with DNA barcodes. Nat Biotechnol.

[29] Xu Q, Schlabach MR, Hannon GJ, Elledge SJ (2009). Design of 240,000 orthogonal 25mer DNA barcode probes. Proc Natl Acad Sci USA.

[30] Kivioja T, Vähärautio A, Karlsson K (2011). Counting absolute numbers of molecules using unique molecular identifiers. Nat Methods.

[31] Andersen RS, Thrue CA, Junker N (2012). Dissection of T-cell antigen specificity in human melanoma. Cancer Res.

[32] McGranahan N, Furness AJS, Rosenthal R (2016). Clonal neoantigens elicit T cell immunoreactivity and sensitivity to immune checkpoint blockade. Science.

[33] Linnemann C, van Buuren MM, Bies L (2015). High-throughput epitope discovery reveals frequent recognition of neo-antigens by CD4^+^ T cells in human melanoma. Nat Med.

[34] Kreiter S, Vormehr M, van de Roemer N (2015). Mutant MHC class II epitopes drive therapeutic immune responses to cancer. Nature.

[35] Roep BO, Peakman M (2011). Diabetogenic T lymphocytes in human type 1 diabetes. Curr Opin Immunol.

[36] Hohlfeld R, Dornmair K, Meinl E, Wekerle H (2015). The search for the target antigens of multiple sclerosis, part 1: autoreactive CD4^+^ T lymphocytes as pathogenic effectors and therapeutic targets. Lancet Neurol.

[37] Crawford F, Kozono H, White J (1998). Detection of antigen-specific T cells with multivalent soluble class II MHC covalent peptide complexes. Immunity.

[38] Vollers SS, Stern LJ (2008). Class II major histocompatibility complex tetramer staining: progress, problems, and prospects. Immunology.

[39] Braendstrup P, Justesen S, Osterbye T (2013). MHC class II tetramers made from isolated recombinant α and β chains refolded with affinity-tagged peptides. PLoS One.

[40] Day CL, Seth NP, Lucas M (2003). Ex vivo analysis of human memory CD4 T cells specific for hepatitis C virus using MHC class II tetramers. J Clin Investig.

[41] Rudensky AYu, Preston-Hurlburt P, Hong SC (1991). Sequence analysis of peptides bound to MHC class II molecules. Nature.

[42] Cole DK, Pumphrey NJ, Boulter JM (2007). Human TCR-binding affinity is governed by MHC class restriction. J Immunol.

[43] Andreatta M, Karosiene E, Rasmussen M (2015). Accurate pan-specific prediction of peptide-MHC class II binding affinity with improved binding core identification. Immunogenetics.

[44] Snyder A, Makarov V, Merghoub T (2014). Genetic basis for clinical response to CTLA-4 blockade in melanoma. N Engl J Med.

[45] Rizvi NA, Hellmann MD, Snyder A (2015). Cancer immunology. Mutational landscape determines sensitivity to PD-1 blockade in non-small cell lung cancer. Science.

[46] Hugo W, Zaretsky JM, Sun L (2016). Genomic and Transcriptomic features of response to anti-PD-1 therapy in metastatic melanoma. Cell.

